# Role of actin filaments and cis binding in cadherin clustering and patterning

**DOI:** 10.1371/journal.pcbi.1010257

**Published:** 2022-07-08

**Authors:** Qilin Yu, Taeyoon Kim, Vijay Rajagopal

**Affiliations:** 1 Department of Biomedical Engineering, University of Melbourne, Melbourne, Australia; 2 Weldon School of Biomedical Engineering, Purdue University, West Lafayette, Indiana, United States of America; University of Pittsburgh, UNITED STATES

## Abstract

Cadherins build up clusters to maintain intercellular contact through trans and cis (lateral) bindings. Meanwhile, interactions between cadherin and the actin cytoskeleton through cadherin/F-actin linkers can affect cadherin dynamics by corralling and tethering cadherin molecules locally. Despite many experimental studies, a quantitative, mechanistic understanding of how cadherin and actin cytoskeleton interactions regulate cadherin clustering does not exist. To address this gap in knowledge, we developed a coarse-grained computational model of cadherin dynamics and their interaction with the actin cortex underlying the cell membrane. Our simulation predictions suggest that weak cis binding affinity between cadherin molecules can facilitate large cluster formation. We also found that cadherin movement inhibition by actin corralling is dependent on the concentration and length of actin filaments. This results in changes in cadherin clustering behaviors, as reflected by differences in cluster size and distribution as well as cadherin monomer trajectory. Strong cadherin/actin binding can enhance trans and cis interactions as well as cadherin clustering. By contrast, with weak cadherin/actin binding affinity, a competition between cadherin-actin binding and cis binding for a limited cadherin pool leads to temporary and unstable cadherin clusters.

## Author summary

Cadherin molecules are transmembrane proteins, which physically link adjacent cells to form tissues. They do so by clustering together laterally in the plasma membrane of each adjoining cell and at the same time associating with cadherin molecules on the opposing cell. These lateral and trans- cadherin interactions are affected by the dynamics and structure of the actin cytoskeleton underlying the plasma membrane of the adjoining cells. In this work, we constructed a biophysics-based computational model to study the effect of the nature of interactions between cadherin molecules and actin filaments on cadherin clustering and cell-cell adhesion formation. Our model suggests that when only cadherin-cadherin interactions are simulated, weak lateral binding between cadherin molecules leads to formation of large cadherin clusters. This counterintuitive finding reconciles a number of apparently contradicting prior experimental observations. When including cadherin-actin filament interactions, the model predicts that the actin network facilitates or inhibits cadherin clustering in different situations that depends on the properties of the actin network and adaptor proteins for cadherin/actin linking. Our model provides an important conceptual framework to mechanistically explain experimental observations that study various cadherin clustering behaviors regulated by the actin cytoskeleton at cell-cell adhesion junctions.

## Introduction

Adherens junctions (AJs) are protein complexes at cell-cell adhesion, that physically link adjacent cells through extracellular binding between cadherin molecules and cytoplasmic binding between AJs and the actin cytoskeleton underlying the cell membrane. Since adhesive activity of individual cadherin molecules is negligible, clustering of cadherin is critical for AJ assembly. Cooperative interactions between extracellular (EC) domains of cadherins, referred to as trans and cis (lateral) interactions, are important for cadherin clustering [1,2]. In addition, the actin cytoskeleton beneath the cell membrane regulates cadherin clustering by corralling and tethering through linking molecules [3]. Actin cytoskeletal network morphology and dynamics also affect the assembly and stability of cadherin clusters [4–8]. A mechanistic understanding of the individual and integrative effects of these different factors on cadherin clustering is lacking. Here we introduce several key gaps in our understanding of cadherin clustering behavior and patterning that we set out to address in this study.

There is a discrepancy between the theoretical prediction and experimental observations of how cis interactions determine cadherin cluster size. It was experimentally shown that the cis interaction is necessary for cell-cell junction formation, and mutations inhibiting the cis interaction lead to impaired adhesion [9]. Direct investigations of the cis interaction are difficult due to its weak affinity [10–12]; the dissociation constant of cis binding is estimated to be between 1 and 10mM, which is a biologically reasonable range of weak molecular binding [13]. However, micrometer-sized cadherin clusters have been observed in experiments [6], eventhough computational studies have suggested that a higher cis binding affinity is required for large cluster formation [2,14].

Cadherin clustering is also affected by the actin cytoskeleton beneath the cell membrane. The actin cytoskeleton regulates cadherin clustering by corralling or tethering directly to cadherins through linking molecules [3]. There is general agreement that actin filaments (F-actin) stabilize cadherin clusters by linking to cytoplasmic domains of cadherin molecules via linker proteins. The linkage between F-actin and cadherin can facilitate cadherin clustering in a cis-independent manner [5]. Clusters formed by cadherin molecules without F-actin tethering are highly transient and mobile, impairing junction stability [15].

α-catenin and vinculin are known to play a crucial role as linker proteins. These linker proteins are usually inactive due to autoinhibitory intramolecular interactions. Upon activation, they expose cryptic actin binding site and extend their length, allowing them to couple cadherins with the actin cytoskeleton. Upon mechanical stimulation, the lifetime of the vinculin/actin bond and the α-catenin/actin bond vary, showing the nature of catch-slip bonds [4,8]. Due to these mechanosensitive linker proteins, forces are involved with the maintenance and remodeling of AJs. Therefore, we set out to systematically investigate how properties of the linker protein affect F-actin tethered cadherin clustering.

The dynamics of the actin cytoskeleton also affects the assembly and stability of cadherin clustering [4–8]. Lifetime of F-actin at AJs ranges from 10 s up to several minutes [7,16,17]. Studies using fluorescence recovery after photobleaching (FRAP) showed that F-actin linked to cadherin-catenin-complex (CCC) in MDCK cells turns over within seconds. In punctate AJs, the turnover of actin bundle tips occurs within ~20 s, which is much faster than the turnover of actin bundle stalks whose average half-life is ~2 min [7]. It was reported that actin turnover rate affects the spatial patterning of cadherin clusters across cell contact interfaces. Cell-doublet experiments demonstrated that the asymmetric distribution of E-cadherin on the intercellular contact is mainly attributed to a locally different actin turnover rate [15]. In addition, various types of actin structures have been observed near AJs, including horizontally aligned actin bundles in zonula AJs and vertically aligned actin bundles in punctate AJs [18]. Recent experiments observed the existence of branched actin networks with various F-actin orientations at AJs [18]. These indicate that cadherins at AJs physically interact with F-actins at various orientations relative to the cell membrane. This implies that the morphology of the actin cytoskeleton affects cadherin clustering. Motivated by these observations, we set out to investigate the effects of actin turnover rate and actin morphology on cadherin clustering.

We adopted a biophysics-based computational modelling approach to systematically quantify the effects of each of the factors outlined above. Various computational models have provided insights into understanding how cadherin dynamics are determined by factors including cadherin structures, cadherin-cadherin interactions, cellular forces, and membrane environments [2,13,14,19–22]. These studies simulated limited number of cadherin molecules and/or simulated short timescales that limit comparisons of cadherin clustering predictions against experimental observations. In addition, the effects of actin structures and cadherin/F-actin linkers on cadherin clustering and patterning were not considered previously.

**Fig 1:**
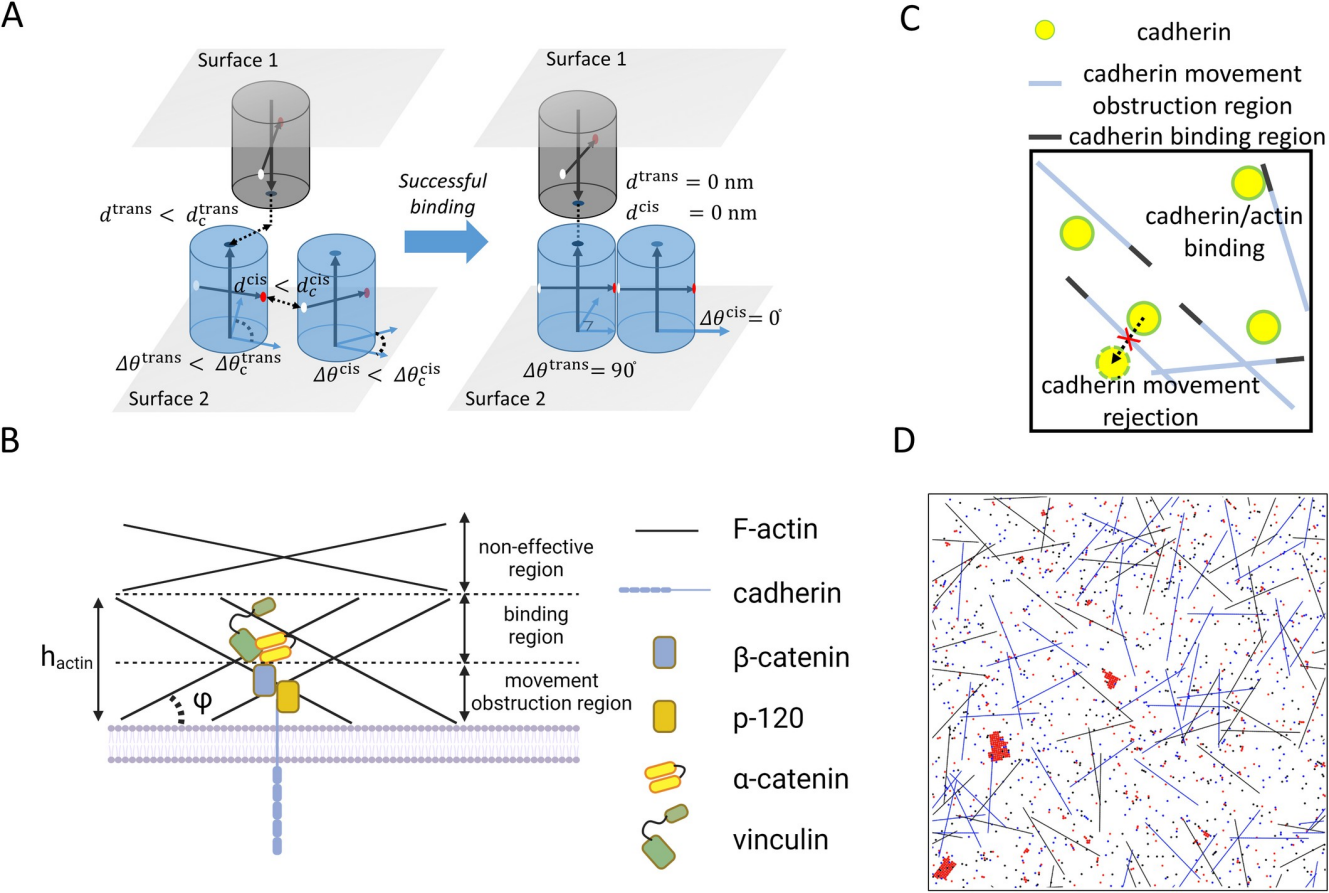
Coarse-grained model for studying cadherin clustering. (A) The intercellular junction is represented by two flat surfaces. The extracellular region of cadherin is simplified as a cylindrical column with 2.5 nm in radius. The trans binding site is located at a center point on the end of the column (dark blue dots). Donor (red dots) and receptor (white dots) sites for cis interactions are located on opposite sides of the lateral surface of the column. The locations of the donor and receptor sites define the orientation of cadherins, *θ* (horizontal arrows). For consideration of trans and cis interactions between two cadherin monomers, a distance between their axes, *d*, and a difference in their orientations, Δ*θ*, are measured. Interactions between two monomers take place if they satisfy two conditions for a distance (*d*<*d*_c_) and an angle (*Δθ*<*Δθ*_c_), where the subscript “c” indicates threshold values. After association, structural adjustment is performed; after trans interactions, the locations of two cadherin monomers are adjusted to make their axes align. After cis interactions, two monomers are slightly relocated to make the distance between their axes equal to the diameter of the columns, and their orientations are synchronized. (B) To simplify three-dimensional actin cortex beneath a membrane, we split the cortex to 3 different regions based on their distance from the membrane: cadherin movement obstruction region, cadherin binding region, and non-effective region. The non-effective region is not considered in our model. (C) Two-dimensional projection of the cadherin movement obstruction region and cadherin binding region. Part of F-actins that belongs to the movement obstruction region limits movement of cadherin monomers by rejecting their movement. Part of F-actins that belongs to the binding region can bind to cadherin monomers if they are sufficiently close to each other. After binding, cadherin monomers stop moving. (D) An example simulation with 1 μm×1 μm area and periodic boundaries. Red dots represent trans dimers, and blue and black dots represent cadherin monomers located on the two surfaces shown in (A). Blue and black line segments represent F-actins located on two surfaces.

We developed an agent-based model of cadherin diffusion and clustering with consideration of the actin cytoskeleton in order to shed light on the physiologically-relevant mechanisms of cadherin clustering (see Fig 1). Our model is able to reproduce experimental observations both quantitatively and qualitatively. We employed the model to probe the sensitivity of the nucleation and growth of AJs to a variation in several potential factors, including the density and cis binding affinity of cadherin, the length, density, and dynamics of F-actin, and the properties of cadherin/actin linkers.

## Methods

We adopted a previous computational model of cadherin-cadherin interactions to study interactions between cadherins and F-actin [14]. The model captures the structural information for the cadherin cluster reflected by x-ray crystallographic data [9,23], which is the basis for assumptions in the model. In our two-dimensional (2D) model, we consider both extracellular and intracellular domains of cadherins and a fraction of the actin network of two adjacent cells by assuming that the 2D model represents the projection of a local three-dimensional (3D) space onto a x-y plane. Cadherin is coarse-grained by an element undergoing diffusion, and F-actin is simplified into a rigid body without movement or deformation. Two plasma membranes at AJ between adjacent cells are represented by two planes located on top of each other in the 3D space, but these two planes coexist in the 2D model (Fig 1D). The x and y dimensions of the planes are 1 μm × 1 μm, and periodic boundary conditions are assumed in x and y directions. Specific details of the cadherin and actin models are outlined below. All parameters and associated references used in the model are available in S1 Table.

### Cadherin model

Cadherins are randomly distributed on the two planes at the beginning of simulations. The extracellular domains of cadherin molecules are represented by a circular monomer whose radius is 2.5 nm in our 2D model (Fig 1A). We assumed that each cadherin contains one trans binding site and two cis binding sites called a donor site and a receptor site. Two monomers that belong to different planes (i.e., membranes) can form a trans-dimer via binding between trans binding sites. Two monomers that belong to the same plane can be linked to each other via the cis-donor site and the cis-receptor site. After two monomers are bound into one trans-dimer, a single cadherin in the trans-dimer is still able to be linked with other monomers or dimers using cis binding sites.

At each time step, cadherins undergo stochastic, brownian motion in the plane where they belong, resulting in diffusion-like movement. They move in a random direction with a constant step size $\sqrt{4D\Delta t}$, where D is a diffusion coefficient, and Δt is time step. Cadherins can also rotate randomly at each time step, but the rotation is restricted along about the membrane normal. We now outline the biophysical underpinnings of the rules that govern cis- and trans- interactions between diffusing cadherin molecules.

A bimolecular reaction representing the trans and cis interactions is described as the following:

$$A+B\overset{k_{+}^{j}}{\underset{k_{-}^{j}}{⇌}}C$$ (1)

where A and B are individual cadherin monomers, and C is a bound complex formed by two cadherin monomers. $k_{+}^{j}$ and $k_{-}^{j}$ are association and dissociation rates of the reaction j, respectively, where j represents either the trans interaction or the cis interaction. This reaction can be separated into a diffusion process and an intrinsic reaction process:

$$A+B\overset{k_{\mathrm{diff}}^{j}}{\underset{k_{\mathrm{diff},b}^{j}}{⇌}}A\cdot B\overset{k_{\mathrm{ass}}^{j}}{\underset{k_{\mathrm{dis}}^{j}}{⇌}}C$$ (2)

where $k_{diff}^{j}$ is a diffusion rate, and $k_{ass}^{j}$ and $k_{dis}^{j}$ are intrinsic association and dissociation rates, respectively. A·B indicates a state where A and B can bind to each other through the intrinsic reaction because the following two conditions are satisfied via diffusion. First, a distance between binding sites of two particles is smaller than the cutoff distance, *d*_c_. *d*_c_ is 1.5 nm and 3 nm for trans and cis interactions, respectively. Second, a difference in the orientation angles of two monomers is smaller than the cutoff angle, *a*_c_ = 30° [13].

The intrinsic association probability, $p_{ass}^{j}$, can be calculated as follows:

$$p_{\mathrm{ass}}^{j}=1-\exp(-k_{\mathrm{ass}}^{j}\Delta t)$$ (3)

After successful association, monomers are aligned structurally with a packing angle of 90° for the trans interaction (*θ*_trans_) and with a packing angle of 180° for the cis interaction (*θ*_cis_) (Fig 1A). In our model, two monomers that are associated within a trans-dimer can diffuse together. By contrast, after forming a cis bond, two cadherins in the complex will be immobilized and stop diffusing [13]. The trans and cis bonds can break with a probability, $p_{dis}^{j}$, calculated from $k_{dis}^{j}$ using Eq 3.

### Model of the actin network and its interaction with cadherins

We created a 2D model of actin-cadherin interaction domains by simplifying the three-dimensional (3D) actin cortex beneath the membrane into an actin network composed of line segments. Each line segment represents the projection of an actin filament existing in a 3D space onto a cell membrane located in a 2D space (Fig 1B–1D). Each segment contains two regions: a cadherin movement obstruction region and a cadherin binding region (Fig 1B). The movement of cadherin is rejected if it tries to cross over the obstruction region of actin filaments (Fig 1B and 1C). A fraction of F-actin is assumed to be the cadherin-binding region where cadherin monomers can bind. As the actin line segments represent the projection of F-actin onto a 2D plane, the length of line segments, *L*_F_, in our model is affected by two factors: i) an angle between the orientation of F-actin and the direction normal to the plane, *φ*, and ii) the thickness of the cadherin effective region which includes only cadherin-diffusion obstruction region or both cadherin-diffusion obstruction and cadherin-binding regions, *h*_actin_ (Fig 1C). Using the two factors, we calculate F-actin length, *L*_F_, in the 2D model as follows:

$$L_{F}=h_{\mathrm{actin}}\tan\varphi$$ (4)

For simplicity, we implicitly considered the linker proteins in the model by assuming that cadherin is directly bound to F-actin. The properties of linker proteins were represented by three parameters defining the cadherin-F-actin bond: the intrinsic association rate, $k_{ass}^{cad/actin}$; the intrinsic dissociation rate, $k_{dis}^{cad/actin}$; and the ratio of the cadherin-binding region. The ratio of the cadherin-binding region is affected by how deeply cadherins can reach within the cytoplasm. For example, since the C-terminus of vinculin can reach F-actin located farther away from the cell membrane, activated vinculin can enable cadherins to bind to a larger pool of F-actin beneath the cell membrane [24], increasing the ratio of the cadherin-binding region.

When a distance between cadherins and the cadherin-binding region of F-actin is smaller than the cutoff distance, *d*_c_ = 3.5 nm, they are eligible for binding with each other (Fig 1B and 1C). Note that 3.5 nm is equivalent to the approximate radius of F-actin [25]. While cadherins are immobilized after binding to F-actin, we assumed that they can still rotate freely since the binding site for F-actin is located inside the membrane, whereas the cis and trans binding sites are located outside the membrane.

## Results

### Weak cis binding affinity facilitates the formation of large cadherin clusters

**Fig 2:**
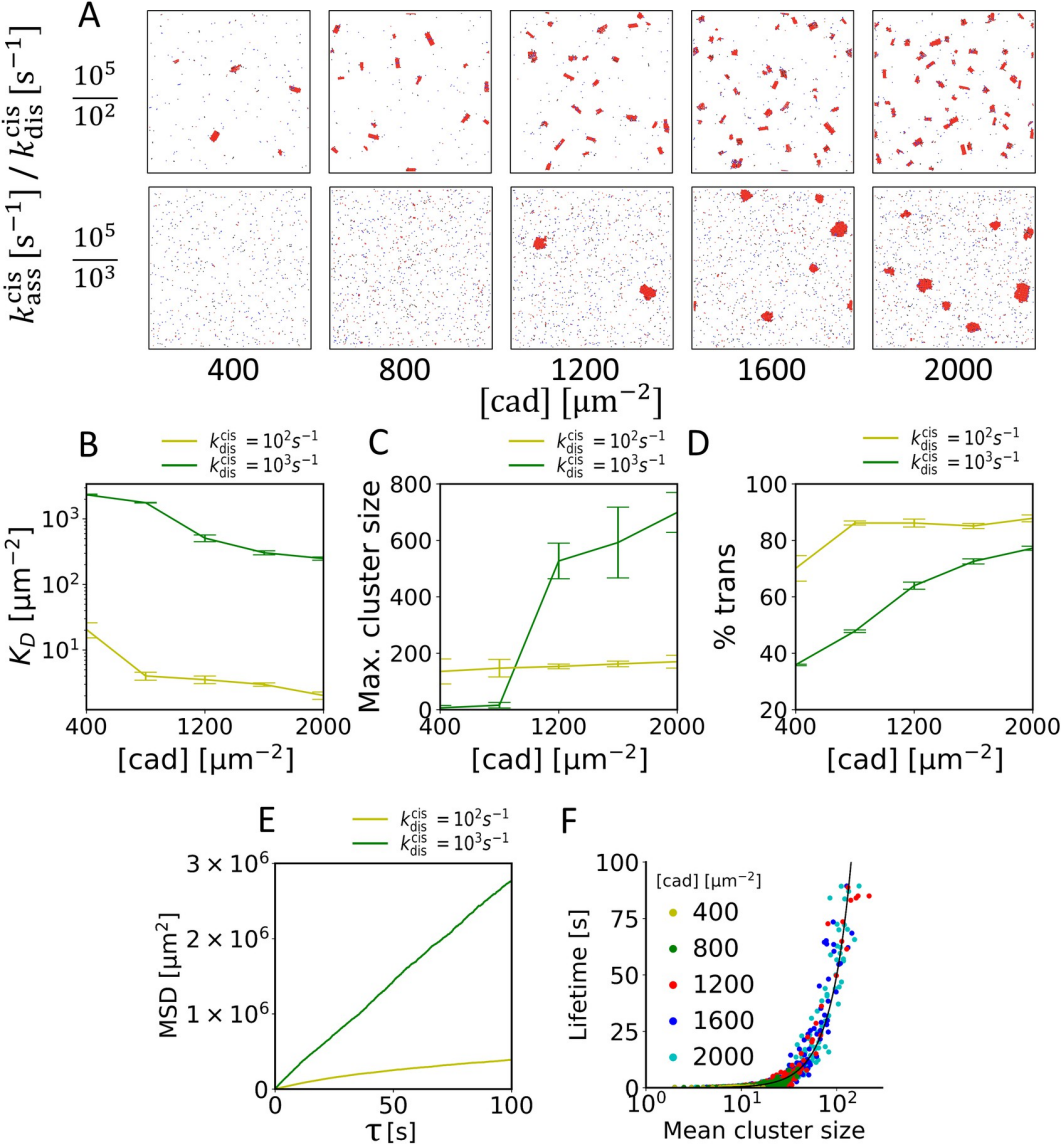
Weak cis binding affinity leads to formation of larger clusters. (A) Snapshots of simulations at 100 s with $k_{ass}^{cis}/k_{dis}^{cis}$ = 1000 (1^st^ row) and 100 (2^nd^ row) and different cadherin concentration, [cad]. (B-D) Comparisons of (B) the cis dissociation constant $K_{D}^{cis}$, (C) maximum cluster size at 100s, and (D) the fraction of cadherin molecules existing as trans dimer measured in simulations shown in (A). (E) Mean squared displacement (MSD) of cadherin monomers measured in two simulations shown in the rightmost column of (A) [cad] = 2000 μm^-2^. Each data point in (B-E) represents the average of data obtained in 5 simulations performed under the same condition. (F) Lifetime of clusters measured in all 5 simulations shown in 2^nd^ row of (A). A solid black line indicates a quadratical increase in the cluster lifetime, ~[cad]^2^. The cluster size is defined as the number of monomers in a cluster.

To investigate the role of cis binding on cluster formation, we tested a wide range of cadherin concentrations, intrinsic association rates, $k_{ass}^{cis}$, and intrinsic dissociation rates, $k_{dis}^{cis}$ (S1 and S2 Figs). $k_{ass}^{cis}$ was varied between 10 and 10^5^, and $k_{dis}^{cis}$ was set to satisfy $k_{ass}^{cis}/k_{dis}^{cis}$ = 1000 (Figs 2A, 1st row and S1) or 100 (Figs 2A, 2nd row and S2). Simulations were run until the number of trans and cis binding events in each simulation converged to a threshold value. We analyzed the dissociation constant of cis, $K_{D}^{cis}$ (Fig 2B), maximum cluster size (Fig 2C), and the percent of cadherins existing as trans-dimers (Fig 2D). Full analysis with other values of $k_{ass}^{cis}$ and $k_{dis}^{cis}$ can be found in S1 and S2 Figs. In general, with higher cadherin concentration, $K_{D}^{cis}$ decreases, and the maximum cluster size and the percent of trans-dimer cadherins increase. Interestingly, a 10-fold increase in $k_{dis}^{cis}$ led to weaker cis binding affinity $K_{D}^{cis}$, but it results in the formation of larger cadherin clusters if cadherin concentration is high enough. Note that maximum cluster sizes obtained with $k_{ass}^{cis}/k_{dis}^{cis}$ = 100 are comparable to previous experimental observations [6]. Also, the $K_{D}^{cis}$ calculated in our simulations falls within the reasonable range for EC1-EC1 mediated dimerization of ~500 μm^-2^ [26]. Our results therefore counter-intuitively imply that weak cis binding affinity is required for large cluster formation.

To further explore the mechanism of large cluster formation induced by weak cis binding affinity, we examined cadherin diffusion, cluster size, and cluster lifetime. As shown by the mean square displacement (MSD) measured using all cadherin molecules, cadherin molecules with high $k_{dis}^{cis}$ show high mobility because a large fraction of the cadherin molecules exist as monomers (Fig 2E). By contrast, in cases with smaller $k_{dis}^{cis}$, the mobility of cadherin molecules is much lower since most of the cadherin molecules belong to clusters. The scatter plot of cluster lifetime vs average cluster lifetime shows that as more cadherin molecules aggregate into a cluster, cluster lifetime increases quadratically (Fig 2F).

If cis binding is weak with high $k_{dis}^{cis}$, a small cluster formed by a few cadherin molecules is likely to be disassembled. Because the association rate $k_{+}^{cis}$ is proportional to not only $k_{ass}^{cis}$ but also cadherin concentration, a small portion of the small clusters can survive and evolve into a larger cluster with higher cadherin concentration. Note that even with the highest cadherin concentration tested here, the association rate is slightly greater than the dissociation rate. These clusters behave as a stable seed where neighboring cadherin monomers with high mobility (i.e., high $k_{diff}^{cis}$) keep binding and unbinding, leading to a slow increase in cluster size over time. As more cadherin molecules aggregate into clusters, cadherin molecules trapped near the cluster center lose freedom of movement, resulting in a significant decrease in $k_{diff,b}^{cis}$ and thus $k_{-}^{cis}$. With these mechanisms, cadherin molecules can form large clusters even though cis binding is weak.

### F-actin induces sub-diffusion of cadherins via local corralling

Having examined impact of cis-interactions on cadherin clustering, we then introduced F-actin-cadherin interactions into the model to study the role of F-actin in cadherin clustering. It has been suggested that the actin cytoskeleton beneath the cell membrane regulates cadherin dynamics by corralling (i.e., surrounding) and tethering the cytoplasmic domain of cadherins through linker proteins [3]. We investigated how the length and density of F-actin regulate cadherin clustering by obstructing cadherin diffusion. To simulate F-actin-induced diffusion obstruction, any movement of cadherins across line segments was rejected. We explored a wide range of F-actin length (200–1000 μm), and F-actin concentration (60–240 μM), which roughly cover the biological reasonable range of F-actin length from 100 μm to several microns [27,28] and concentration from 46–70 μM in non-muscle cells to 230–960 μM in muscle cells [29]. We assumed that the cadherin effective region includes only actin meshwork obstructing cadherin movement. Thus, the thickness of the region was assumed to be *h*_actin_ = 100 nm, which is approximately the deepest distance that EPLIN can reach out beneath the cell membrane [24]. It was assumed that $k_{ass}^{cis}$ and $k_{dis}^{cis}$ are 10^5^ s^-1^ and 10^3^ s^-1^, respectively.

**Fig 3:**
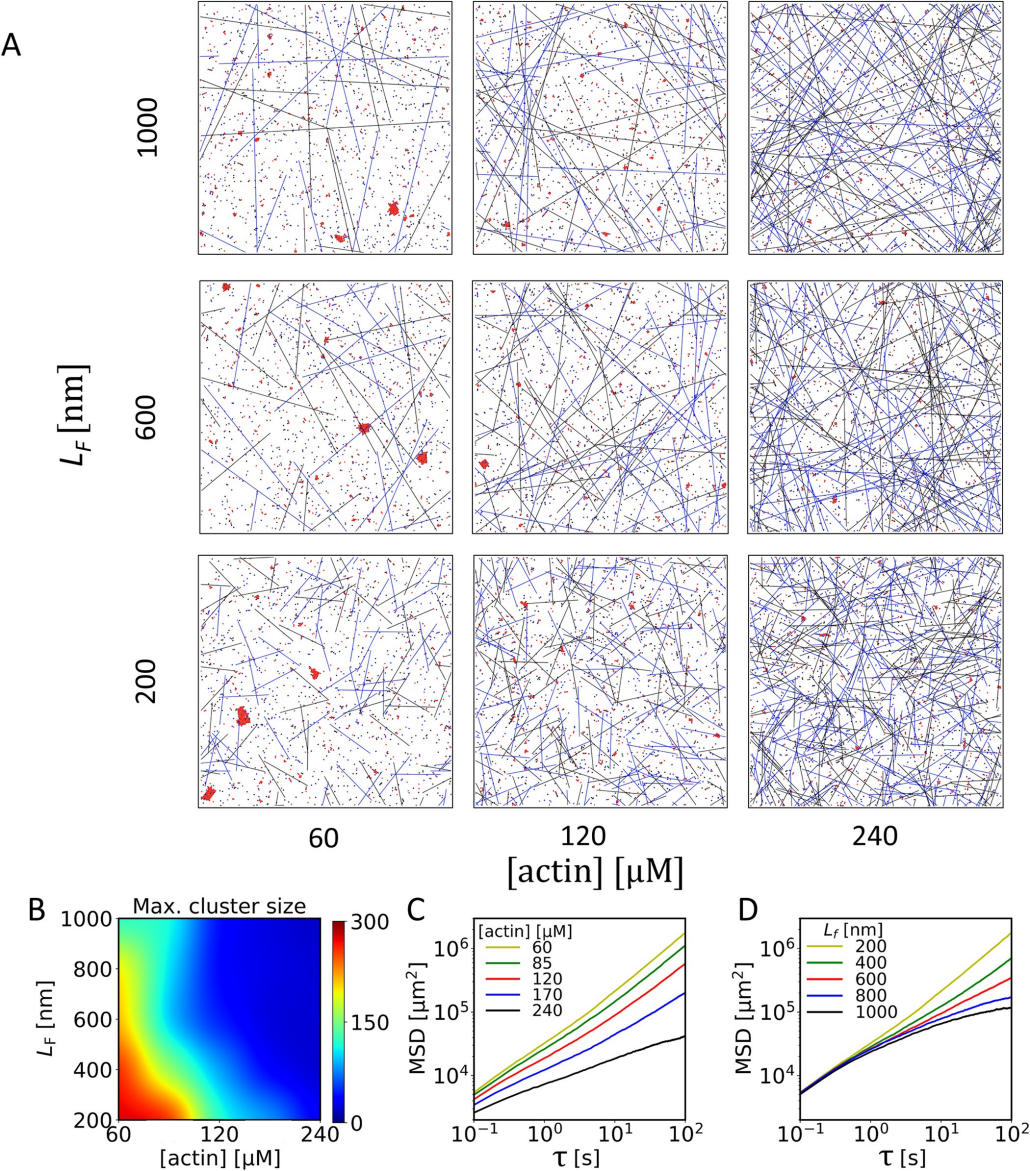
The presence of F-actin reduces the diffusivity of cadherins. (A) Snapshots of simulations at 100 s with a range of actin concentrations, [actin], and F-actin length, *L*_F_, with $k_{ass}^{cis}=10^{5}\;s^{-1},\;k_{dis}^{cis}=10^{3}\;s^{-1}$, [cad] = 1200 μm^-2^. (B) Maximum size of clusters as a function of actin concentration and F-actin length. Clusters tend to be smaller with higher actin concentration and F-actin length. (C) Mean squared displacement (MSD) of cadherin molecules with different actin concentrations and *L*_F_ = 200 nm. As actin concentration increases, the magnitude and slope of MSD were reduced, which is attributed to smaller meshes formed by F-actins. The consistently lower slope with higher actin concentration indicates sub-diffusive motions. (D) MSD with different F-actin length and [actin] = 60 μM. With longer F-actins, F-actin forms closed meshes, which confines cadherin movement only at a later time point. Each data point in (B-D) is the average of data obtained in 5 simulations performed under the same condition.

The maximum cluster size decreased with higher F-actin concentration or F-actin length (Fig 3A and 3B). The percent of cadherins existing as trans-dimers or cis-dimers showed a very similar pattern to the cluster size (S3A and S3B Fig). This suggests that trans and cis interactions were largely inhibited with higher F-actin concentration or F-actin length due to corralling by F-actins, which resulted in the formation of smaller clusters. We also analyzed MSD using all cadherins with different F-actin concentrations and lengths (Fig 3C and 3D). When F-actins were short and sparsely distributed, MSD was relatively high because F-actins under such a condition could not obstruct movements of cadherins effectively as can be seen in the trajectories of cadherins (Fig 3E). When F-actin concentration became larger, the magnitude and slope of MSD decreased at all time points (Fig 3C). As there are more F-actin segments, the mesh size of the actin network decreases (Fig 3A). Therefore, cadherins repeat moving within a smaller mesh and then slip away to adjacent meshes (S3D Fig), leading to the decrease in both magnitude and slope of MSD for all time lags. By contrast, when varying F-actin length, MSD was almost identical at small time lags, whereas the magnitude and slope of MSD were lower at long time lags (Fig 3D). Average mesh size is not affected significantly by a change in F-actin length, so MSD at short time lags did not change noticeably and showed a slope close to 1, indicative of diffusive motions (Fig 3C). However, as filament length increases, longer F-actin segments form many “closed” meshes within which cadherins keep moving (S3E Fig). Because those cadherins could not hop to other meshes, the magnitude and slope of MSD became much lower at long time lags. In sum, an increase in both length and concentration of F-actin results in sub-diffusive motions of cadherins at long-time scales, but an increase in only F-actin concentration can induce sub-diffusive motions at short-time scales.

### Properties of linker proteins determine cadherin clustering behaviors

**Fig 4:**
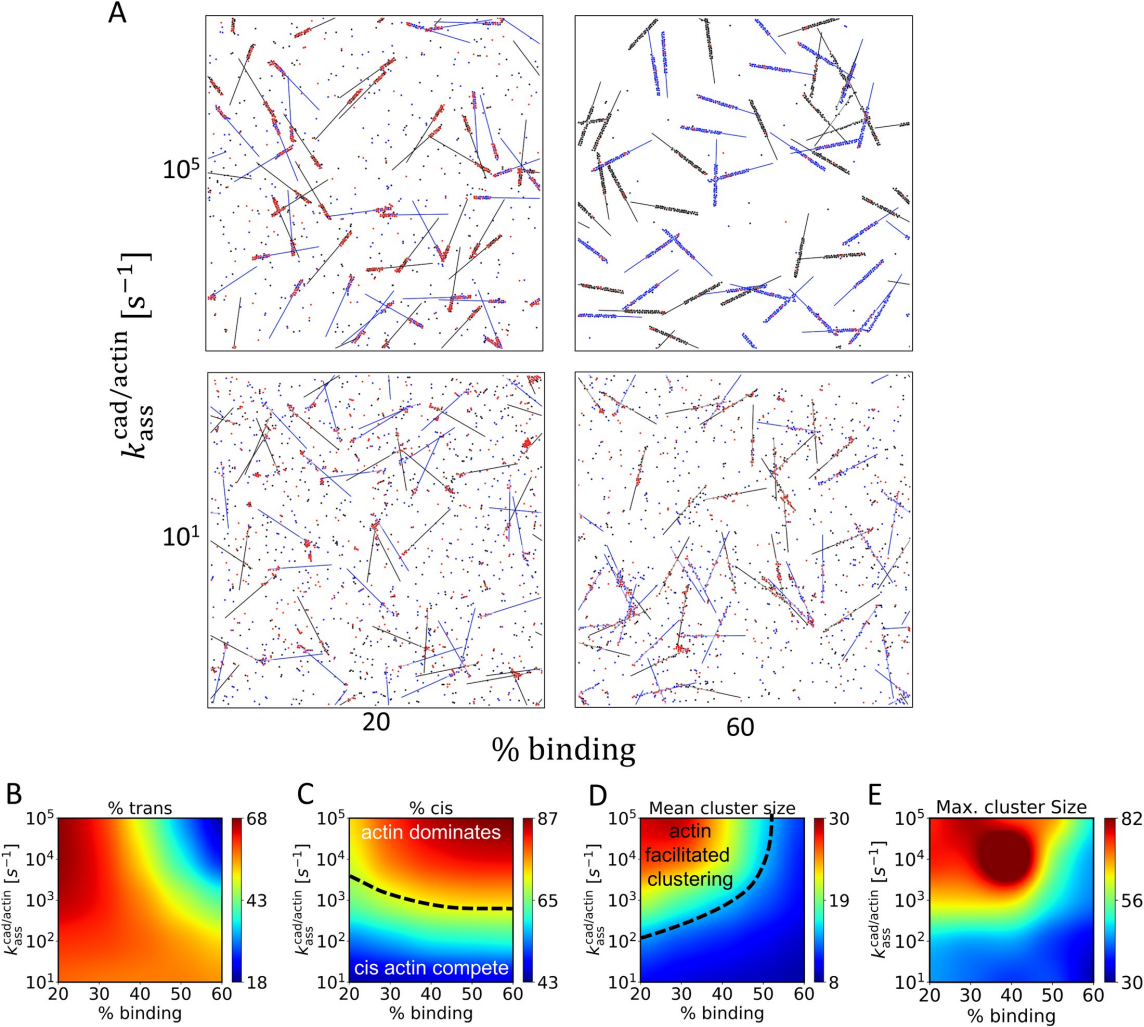
Cooperation and competition between F-actin binding and cis binding mediate cadherin clustering. (A) Snapshots of simulations at 100 s with two different association rate between F-actin and cadherin, $k_{ass}^{cad/actin}$, and two different percents of cadherin-binding region on F-actin. We used $k_{ass}^{cis}=10^{5}\;s^{-1},\;k_{dis}^{cis}=10^{3}\;s^{-1}$, $[cad]=1200\;{\mu m}^{-2},\;k_{dis}^{cad/actin}=0.1\;s^{-1}$. (B-D) With variations in $k_{ass}^{cad/actin}$ and the fraction of binding region, we quantified (B) the fraction of cadherin molecules involved with trans dimer, (C) the fraction of cadherin molecules involved with cis interactions, (D) mean size of clusters formed by more than 4 cadherin monomers, and (E) maximum cluster size.

Binding between cadherin and F-actin is formed through linker proteins, such as vinculin, α-catenin, and EPLIN. We investigated how the properties of these linker proteins affect cadherin clustering. Two sets of simulations were performed with high ($k_{dis}^{cad/actin}$ = 10 s^-1^, S4A Fig) or low intrinsic dissociation rates ($k_{dis}^{cad/actin}$ = 0.1 s^-1^, Fig 4A), to cover the range of cadherin-actin bond lifetime when the bond is adapted via vinculin and α-catenin respectively [4,8]. By calculating percentage of the distribution overlap between α-catenin/actin and vinculin(N-terminus)/actin in an experimental study of the protein organization in cadherin-based adhesions [24], we estimated a range from 20% to 60%, and used as % binding region in this study. $k_{ass}^{cis}$ and $k_{dis}^{cis}$ were fixed at 10^5^ s^-1^ and 10^3^ s^-1^. Since our model does not account for linker proteins explicitly, we instead explored a wide range of the intrinsic association rate $k_{ass}^{cad/actin}$. We also changed the fraction of cadherin-binding region to probe the influences of different depths to which the linker proteins can reach out beneath the contact surface.

First, we set the percent of cadherin-binding region at 20%. With a low intrinsic association rate $k_{ass}^{cad/actin}$ = 10 s^-1^, cadherin recruitment by F-actin could not dominate the cis binding (Fig 4). Cadherin-actin and cis binding compete for a limited cadherin pool, leading to the formation of small, unstable clusters near F-actin and lower fraction of cis binding (Fig 4A, 4C, and 4D). Note that the size of these clusters is noticeably smaller than simulations without F-actin with the same values of $k_{ass}^{cis}$ and $k_{dis}^{cis}$ (c.f., Fig 2A). As we increased $k_{dis}^{cad/actin}$ to 10 s^-1^, this tendency became more obvious (S4C Fig and S1–S3 Movies). With an even weaker cadherin/F-actin binding, cis binding dominates cadherin clustering. Cadherin form large clusters independent of actin as the clusters observed in Fig 2A (S4A and S4D Fig). The percent of the trans binding was higher, whereas the percent of the cis binding was similar (S4B and S4C Fig). Thus, with low affinity between F-actin and cadherin, cadherin clustering was not affected significantly by cadherin-actin binding. Due to the negligible role of the cadherin-actin binding, a change in the fraction of the cadherin-binding region does not cause a noticeable change in the cadherin clustering (Fig 4A–4D).

As the intrinsic association rate $k_{ass}^{Cad/Actin}$ increases to 10^5^ s^-1^, both cadherin-actin binding and cis binding cooperate to anchor a large fraction of cadherins directly to actin filaments. The proximity of cadherins bound to the same F-actin enables the cis interaction to happen more frequently (Fig 4C). High affinity of cadherin-actin binding immobilizes cadherins when they are not involved with cis interactions, effectively decreasing $k_{diff,b}^{cis}$. As a result, the percent of cis interactions was significantly higher than that in the presence of F-actin without cadherin-actin binding (c.f., S3B Fig). Due to a small fraction of cadherin binding sites on F-actin (20%), recruited cadherins can undergo trans dimerization better (Fig 4B), so clusters formed under this condition are large (Fig 4D).

However, this cooperation between cadherin-actin binding and cis binding did not always have positive effects on cadherin clustering. When we increased the fraction of the cadherin-binding region on F-actin from 20% to 50% or above with $k_{ass}^{cad/actin}$ = 10^5^ s^-1^, the percent of the trans interaction decreased substantially, whereas the percent of the cis interaction increased even more (Fig 4A–4C). Most of the individual monomers were sparsely recruited to large binding sites on F-actins on both sides of the intercellular junction. The depletion of free cadherin monomers prevented trans dimerization from occurring well. Cadherins tended to form linear structure via cis interactions along F-actin as observed in previous experiments [5]. Even with larger $k_{dis}^{cad/actin}$ = 10 s^-1^, the dependence of the percent of the trans and cis bindings and cluster size on the fraction of cadherin-binding region was similar (S4B–S4D Fig).

In sum, cadherin-actin binding facilitates or inhibits trans and cis interaction depending on conditions, by localizing cadherins near F-actin. Low binding affinity between F-actins and cadherins did not cause a substantial difference in the cadherin clustering. High binding affinity or larger binding sites on F-actin always increased the frequency of the cis binding. Trans binding can occur frequently if cadherins bind to small binding sites on F-actin with high affinity, and clusters become relatively large. Our result further confirmed that disruption in either trans or cis hurts cadherin clustering and junction adhesiveness.

### The architecture and turnover of the actin cytoskeleton affect cadherin clustering

**Fig 5:**
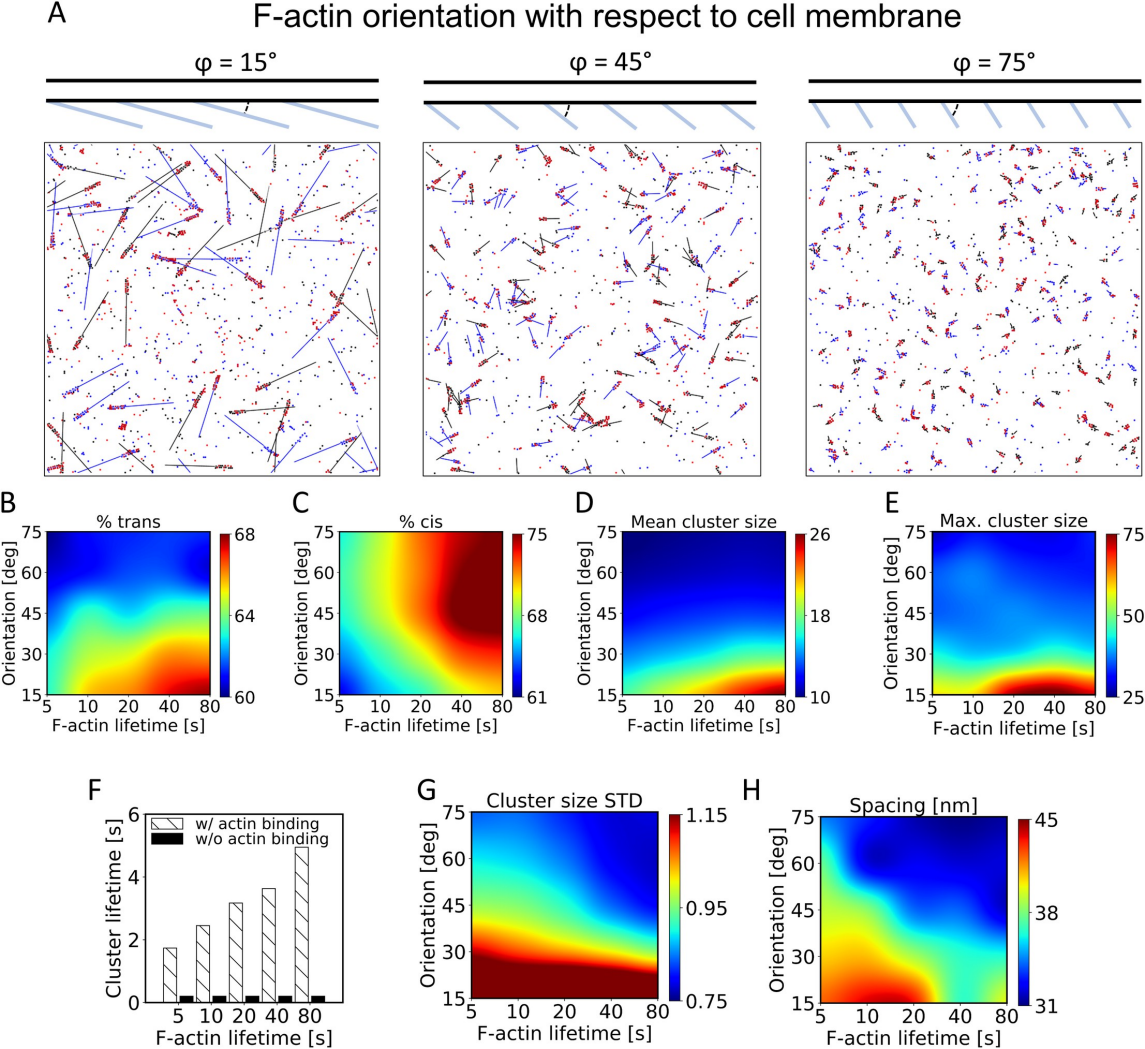
The structure and turnover of F-actins highly affects cadherin clustering. (A) Snapshots of simulations at 100 s with different orientations of F-actins shown on the top. For these simulations, we used $k_{ass}^{cis}=10^{5}\;s^{-1},\;k_{dis}^{cis}=10^{3}\;s^{-1}$, $[cad]=1200\;{\mu m}^{-2}$, $k_{dis}^{cad/actin}=0.1\;s^{-1}$, $k_{ass}^{cad/actin}=10^{5}\;s^{-1}$. (B-D) With variations in the orientation and lifetime of F-actins, we measured (B) the fraction of cadherin molecules existing as trans dimer, (C) the fraction of cadherin molecules involved with cis interactions, (D) mean size of clusters formed by more than 4 cadherin monomers, and (E) maximum cluster size. (F) The mean lifetime of cadherin clusters as a function of F-actin lifetime and F-actin/cadherin binding. Clusters tend to be larger with longer F-actin lifetime and in the presence of the F-actin/cadherin binding. Lifetime of clusters without F-actin binding are not affected by the F-actin lifetime. (G) Standard deviation of cluster size normalized by mean cluster size. (H) The nearest distance between neighboring cadherin clusters.

Considering various orientations and turnover of F-actins at AJs, it is likely that cadherin clustering would be affected significantly by the architecture and dynamics of F-actins. We investigated the effects of F-actin structure and lifetime on cadherin clustering with the assumption of *h*_actin_ = 50 nm. If F-actin is aligned more vertically relative to the membrane, a smaller portion of F-actin would appear in the cadherin effective region (Figs 1C, 1D, and 5A). This can be represented by an even shorter F-actin line segment in our 2D model because less of the vertically aligned filament is projected to the 2D plane. In all simulations, we adjusted the number of F-actins to maintain the concentration of actin included in the 2D model at constant level. The lifetime of F-actin was also varied between 5 and 80 s. We employed $k_{ass}^{cis}$ = 10^5^ s^-1^, $k_{dis}^{cis}$ = 10^3^ s^-1^, $k_{ass}^{cad/actin}$ = 10^5^ s^-1^, $k_{dis}^{cad/actin}$ = 0.1 s^-1^.

We observed that networks aligned perpendicular to the contact interface created more punctate cadherin clusters because only a small region on F-actin is available for interactions with cadherin (Fig 5A). With F-actin aligned more parallel to the membrane, clusters tended to be larger but have more linear shapes. With longer F-actin lifetime, trans and cis interactions were enhanced due to a stable actin network (Fig 5B and 5C). These results agree well with experimental findings that showed F-actin stabilization increases the fraction of overlap between cadherin on two sides of a cell-cell junction [6]. Fig 5D shows that collaboration between trans and cis interactions can increase cluster size, which is in line with results shown in Fig 4 and a previous computational study [2]; the cluster size tended to be larger under conditions leading to the high percent of trans interactions, but the large percent of cis interactions can increase the cluster size too. Not surprisingly, the lifetime of clusters with cadherin-actin binding increased as the lifetime of F-actin increases. By contrast, the lifetime of clusters was independent of F-actin lifetime in the absence of cadherin-actin binding (Fig 5E). We further investigated the effect of F-actin lifetime on the cadherin clustering without cadherin/F-actin binding. Our analysis suggested that dynamic actin network gives cadherin molecules more freedom of movement, enabling more interaction and formation of larger clusters (S5 Fig).

The binding of cadherins to only a small portion of F-actin made the distribution of cluster size more homogeneous (Fig 5F). It also decreased the average of the nearest neighborhood distance between cadherin clusters, meaning that clusters are distributed more uniformly (Fig 5G). Thus, more vertically aligned F-actins result in cadherin clusters with more uniform size and distribution.

## Discussion

In this study, we used a new computational model to show four important factors governing cadherin clustering. First, we showed that weak cis binding affinity promotes the formation of large cadherin clusters. Second, we demonstrated that F-actins can inhibit cadherin clustering by reducing cadherin diffusion. Third, it was shown that the binding of cadherins to F-actins via linker proteins can facilitate cadherin clustering by increasing the percent of the trans and cis interactions, but binding to F-actin can also inhibit cadherin clustering under different conditions. Finally, we suggested that the architecture and lifetime of F-actins affect the size, stability, and patterning of cadherin clusters.

Many studies have shown the necessity of cis interactions for cell-cell adhesions. Computational model studies suggested that the high affinity of cis interactions ($K_{D}^{cis}$ ~ 100 μM, or about 9 *kT*) is required for cluster formation [2,14], which is opposite to our findings. However, cis interactions are too weak to be detected in experiments [10–12]; the affinity of cis dimerization is estimated to be weaker than 1 mM which can be translated to a 2D affinity of 500 *μm*^*-2*^ [26]. Although previous theoretical model predicted that cis binding affinity would be increased dramatically if at least one cadherin molecule in trans is already engaged in a trans-dimer, there is no direct experimental proof supporting the speculation.

By exploring a wide range of binding and unbinding rate of cis interaction (Figs 2, S1, and S2), we showed that cis binding along with trans binding is enough for the formation of micrometer-sized clusters. Weak binding affinity is not a drawback but a benefit for large cluster formation since it allows more interactions between monomers. A large cluster can trap cadherin molecules in its center, locally lowering cadherin diffusivity and thus extending the cluster lifetime.

Most clusters in our model are packed into a crystal lattice structure. However, recent experiments revealed the co-existence of crystal-like packing regions and loose cadherin regions within micrometer-sized adhesion clusters, which is not shown in our model. Based on our computational insights, it is likely that this phenomenon resulted from multiple factors, including non-specific cis interactions [19] and the existence of other proteins on the membrane [13,14]. Incorporating these effects might be helpful to further study cadherin clustering in later studies.

The actin cytoskeleton plays a significant role in regulating cadherin dynamics. Several previous experiments suggested that F-actin corrals cadherin into clusters [3,30,31]. For example, in one of the studies, photoactivated localization microscopy (PALM) was applied to reveal the spatial organization of cadherins and F-actins on the cell-cell contact of the epithelial sheet. Results in this study suggested that cadherin molecules on the contact are loosely organized in small ~50-nm cadherin clusters with F-actin forming fences that obstruct cadherin diffusion [31]. The analysis from our simulation shows that cadherin diffusion is affected by both concentration and length of F-actins (Fig 3). The presence of more F-actins reduced the diffusion of cadherins to sub-diffusion at all time scales due to a decrease in mesh size, whereas longer F-actins resulted in sub-diffusion at only long time scales by forming non-escapable meshes for cadherins which is a novel mechanism emerging from the simulations. The inhibition of diffusive motions of cadherins led to lack of trans and cis interactions, which results in smaller clusters. However, Indra et al. also reported micrometer-size clusters on the basolateral AJs of A431 cells using the same cell line and same microscopy technique [6]. Our results shown in Figs 2F and 3 demonstrate that these observations are not contradictory. Big stable clusters and small transient clusters can co-exist in the same cell.

It has been proposed that binding of cadherins to F-actin guides cadherin cluster assembly [5]. However, a study using single molecule localization microscopy showed that the probability of overlap between cadherins and F-actins measured using Mander’s coefficient is very low, 0.076 [31]. We hypothesize that these contradictory observations result from differences in the properties of cadherin/actin linker proteins. After systematically analyzing simulation data obtained over wide ranges of effective cadherin-actin binding and unbinding rates, we propose that there are three scenarios.

In the first scenario with high cadherin-actin affinity, cadherin binding to F-actin facilitates cis binding by bringing cadherin molecules close to each other, promoting cluster formation. This has been experimentally observed in cadherin-actin links facilitated by α-catenin; the α-catenin actin binding domain (α-ABD) has a high binding affinity of 1μM [5]. In the second scenario, with an intermediate cadherin-actin affinity comparable to affinity between cadherins for cis binding, cis binding and cadherin-actin binding compete for a limited cadherin pool. Cadherin molecules are still being recruited to F-actin, but cadherin molecules can form small clusters in a manner independent of F-actin. In the third scenario with very low cadherin/actin affinity, cadherin clustering is independent of F-actin, and cis binding dominates. In this scenario, cadherin molecules form large clusters (S4A Fig) which are comparable with clusters in Fig 2A. The low cadherin/F-actin binding affinity might be the reason for lack of cadherin-actin overlaps found in the previous experiment [31].

In addition, our simulations showed that an increase in the range of F-actin pool that cadherins can reach out to enable cadherin molecules to form elongated clusters along F-actins. This is consistent with an experimental observation where elongated cadherin clusters along actin bundles were formed when F-actin–binding domain of human utrophin (UtrABD) was used as the cadherin/F-actin binding linker [5]. The same experiment also showed that a UtrABD-based junction is completely absent of filopodia-like protrusions [5]. Our results (Fig 4A and 4B) showed that strong cadherin/actin binding affinity with too many actin binding sites inhibits the trans dimerization of cadherins, which is necessary for inducing actin polymerization and protrusion at the adherens junctions via cSrc-Rac-Arp2/3 signaling pathway [32]. Meanwhile, it is known that arp2/3 is important for initiation and maintenance of filopodia [33]. In conclusion, our finding suggested that trans dimerization inhibited by strong cadherin/actin binding might be the reason for absence of actin dependent protrusion in the experimental study [5].

Recent electron microscopy images of AJs have shown that the structure of the actin cytoskeleton beneath the cell membrane is far more diverse [18]. Arp2/3-generated branched actin networks dominate the earliest phases of contact formation [34,35], whereas actin bundle structures are abundant at mature polarized epithelia [36]. The half-life of AJ-associated F-actin ranges from seconds [37], similar to the lifetime of F-actin in branched actin networks in lamellipodia [38,39], to several minutes [7], similar to the lifetime of actin bundles [40]. Correlating these findings with our results shown in Fig 5, we hypothesize that branched actin networks lead to the formation of smaller, transient but more homogeneously distributed cadherin clusters. This might contribute to the adaptive plasticity of cell adhesion during the early phase of contact formation. However, as the cell adhesion matures, larger and more stable cadherin clusters associated with actin bundles parallel aligned to cell membrane ensure the integrity and stability of cell adhesion.

In conclusion, our simulation results suggest the importance of weak cis binding for cadherin clustering and support a model in which the mechanical and chemical environments in cells determine how important a role F-actin plays in cadherin clustering. F-actin regulates cadherin behaviors by both corralling and tethering. Although the intervention of F-actins during the process of cadherin clustering can inhibit or facilitate trans and cis binding, it will not affect the basic mechanism of cadherin clustering driven by cooperation between trans and cis interactions. Our simulation analyses provide an important conceptual framework to mechanistically unify experimental observations that study various cadherin clustering behaviors regulated by the actin cytoskeleton at AJs.

## Supporting information

S1 FigFull simulation snapshots and simulation analysis for $k_{ass}^{cis}/k_{dis}^{cis}$ equals 1000.(A) Snapshots of simulations at 100 s. Red dots represent trans dimers; blue and black dots represent cadherin monomers on each side. (B) Dissociation constants $K_{D}^{cis}$ of cis binding. (C) Maximum cluster size. (D) The fraction of cadherin molecules existing as trans dimer. Each data point in (B-D) represents the average of data obtained in 5 simulations performed under the same condition.(TIF)

S2 FigFull simulation snapshots and simulation analysis for $k_{ass}^{cis}/k_{dis}^{cis}$ equals 100.(A) Snapshots of simulations at 100 s, $k_{ass}^{cis}/k_{dis}^{cis}$ equals 100. Red dots represent trans dimers; blue and black dots represent cadherin monomers on each side. (B) Dissociation constants $K_{D}^{cis}$ of cis binding. (C) Maximum cluster size. (D) The fraction of cadherin molecules existing as trans dimer. Each data point in (B-D) represents the average of data obtained in 5 simulations performed under the same condition.(TIF)

S3 Fig Trans and cis percent and example trajectories. (A, B) trans and cis ratio for simulations in Fig 3. Each data point in heat maps is an average over 5 simulations. (C-E) Red dots and lines show the examples of cadherin trajectories under three conditions. Time point of the trajectory is indicated by the color scaling. Light red dots indicate early in the time point. Dark red dots indicate latter in the time point. Light gray lines represent actin filaments. (C) *L*_F_ = 200 nm, [actin] = 60 μM, (D) *L*_F_ = 200 nm, [actin] = 240 μM, and (E) *L*_F_ = 1 μm, [actin] = 60 μM. Each data point in (A-B) represents the average of data obtained in 5 simulations performed under the same condition.
(TIF)

S4 FigCooperation and competition between F-actin binding and cis binding on mediate cadherin clustering for higher cad/actin dissociation rate.(A) Snapshots of simulations at 100 s with different F-actin concentration and filament length, $k_{ass}^{cis}=10^{5}\;s^{-1},\;k_{dis}^{cis}=10^{3}s^{-1}$, $[cad]=1200{\mu m}^{-2},\;k_{dis}^{cad/actin}=10\;s^{-1}$. (B) The fraction of cadherin molecules existing as trans dimer. (C) The fraction of cadherin molecules involved in cis interactions. (D) Mean cadherin cluster size for clusters with more than 4 monomers. (E) Maximum cluster size.(TIF)

S5 FigSimulation snapshots and cadherin clustering analysis with various actin lifetime without cad/actin binding.(A) Snapshots of simulations at 100 s, $k_{ass}^{cis}=10^{5}\;s^{-1},\;k_{dis}^{cis}=10^{3}\;s^{-1},\;[cad]=1200\;{\mu m}^{-2}$, *L*_*f*_ = 600 μm, [actin] = 120 μM. Binding between cadherin and F-actin is disabled. (B) The fraction of cadherin molecules existing as trans dimer (green curve) and the fraction of cadherin molecules involved in cis interactions (red curve). (C) Maximum cluster size at 100s. Each curve in (B-C) represents the average of data obtained in 5 simulations performed under the same condition.(TIF)

S1 TableOutlines all the parameters and associated references used in the computational model.The value of $d_{c}^{cad/actin}$ equals half of the diameter of actin filaments [41]. The values of $\phi^{F-actin/memb}$ were selected to cover a wide range of actin orientations. The range of $k_{ass}^{cad/actin}$ is determined arbitrarily due to lack of references.(DOCX)

S1 MovieActin binding dominates cadherin clustering and patterning.$k_{ass}^{cis}=10^{5}\;s^{-1},\;k_{dis}^{cis}=10^{3}s^{-1}$, [cad] = 1200μm^−2^, $k_{ass}^{cad/actin}=10^{5}\;s^{-1},\;k_{dis}^{cad/actin}=10\;s^{-1}$.(MP4)

S2 MovieActin binding and cis binding compete for cadherin clustering and patterning.$k_{ass}^{cis}=10^{5}\;s^{-1},\;k_{dis}^{cis}=10^{3}s^{-1}$, [cad] = 1200μm^−2^, $k_{ass}^{cad/actin}=10^{3}\;s^{-1},\;k_{dis}^{cad/actin}=10\;s^{-1}$.(MP4)

S3 MovieCis binding dominates cadherin clustering and patterning.$k_{ass}^{cis}=10^{5}\;s^{-1},\;k_{dis}^{cis}=10^{3}s^{-1}$, [cad] = 1200μm^−2^, $k_{ass}^{cad/actin}=10\;s^{-1},\;k_{dis}^{cad/actin}=10\;s^{-1}$.(MP4)
