# Supplementary figures and images for: Role of actin filaments and cis binding in cadherin clustering and patterning
Source: PLoS Comput Biol. 2022 Jul 8;18(7):e1010257. doi: 10.1371/journal.pcbi.1010257 (PMC9299298; doi:10.1371/journal.pcbi.1010257)

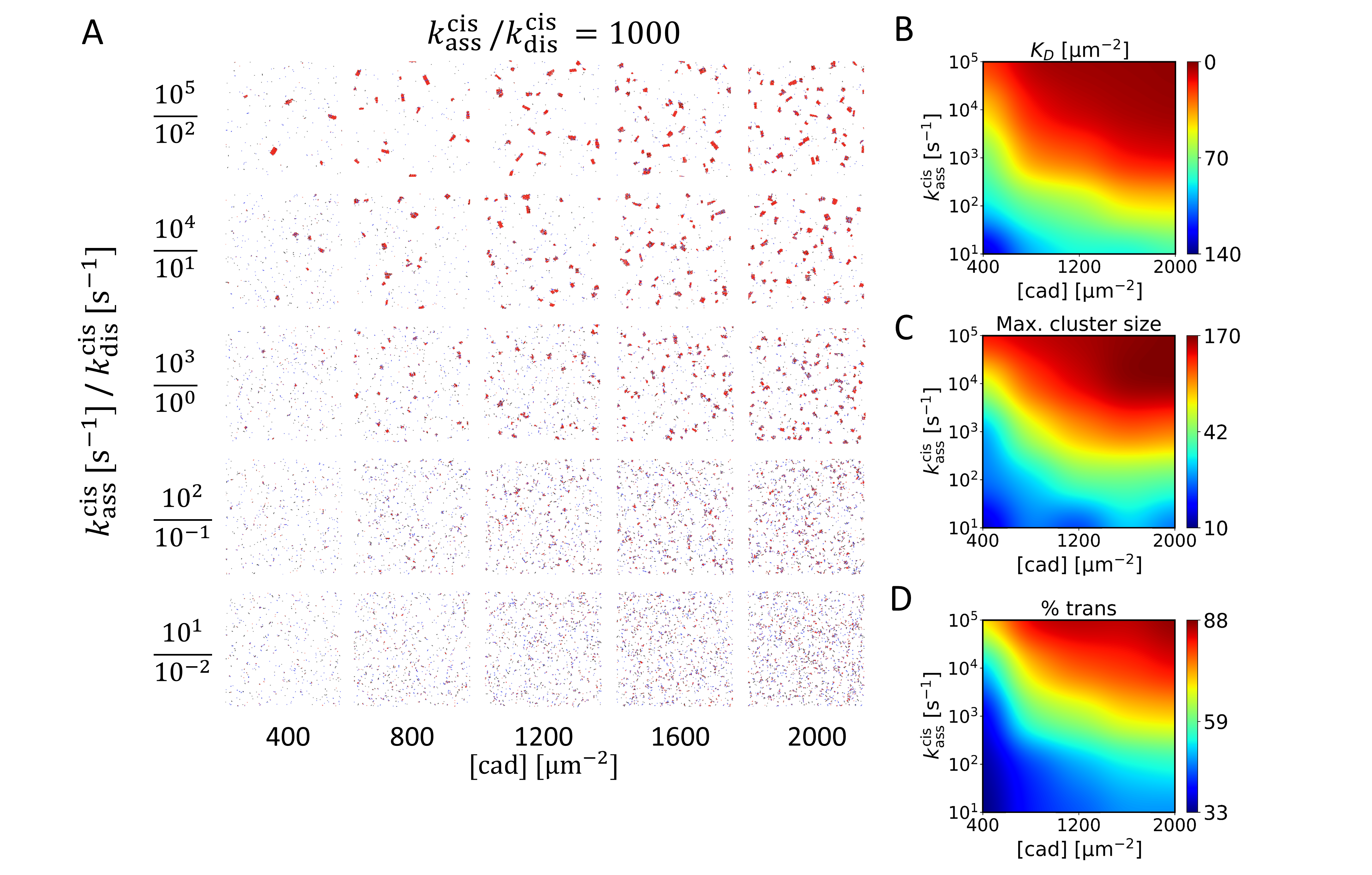

Supplement: S1 Fig — (A) Snapshots of simulations at 100 s. Red dots represent trans dimers; blue and black dots represent cadherin monomers on each side. (B) Dissociation constants KDcis of cis binding. (C) Maximum cluster size. (D) The fraction of cadherin molecules existing as trans dimer. Each data point in (B-D) represents the average of data obtained in 5 simulations performed under the same condition. (TIF) [file pcbi.1010257.s001.TIF]

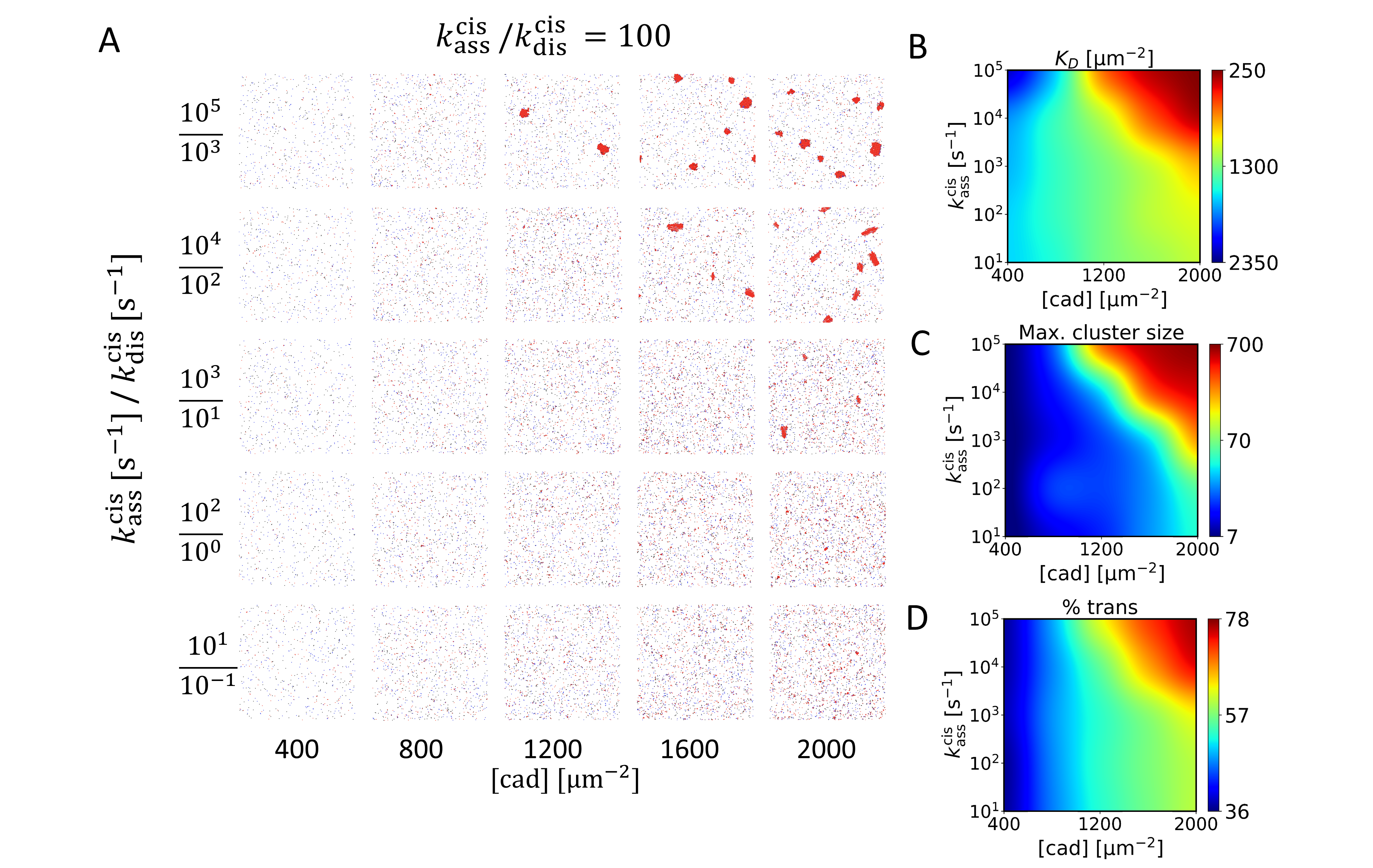

Supplement: S2 Fig — (A) Snapshots of simulations at 100 s, kasscis/kdiscis equals 100. Red dots represent trans dimers; blue and black dots represent cadherin monomers on each side. (B) Dissociation constants KDcis of cis binding. (C) Maximum cluster size. (D) The fraction of cadherin molecules existing as trans dimer. Each data point in (B-D) represents the average of data obtained in 5 simulations performed under the same condition. (TIF) [file pcbi.1010257.s002.TIF]

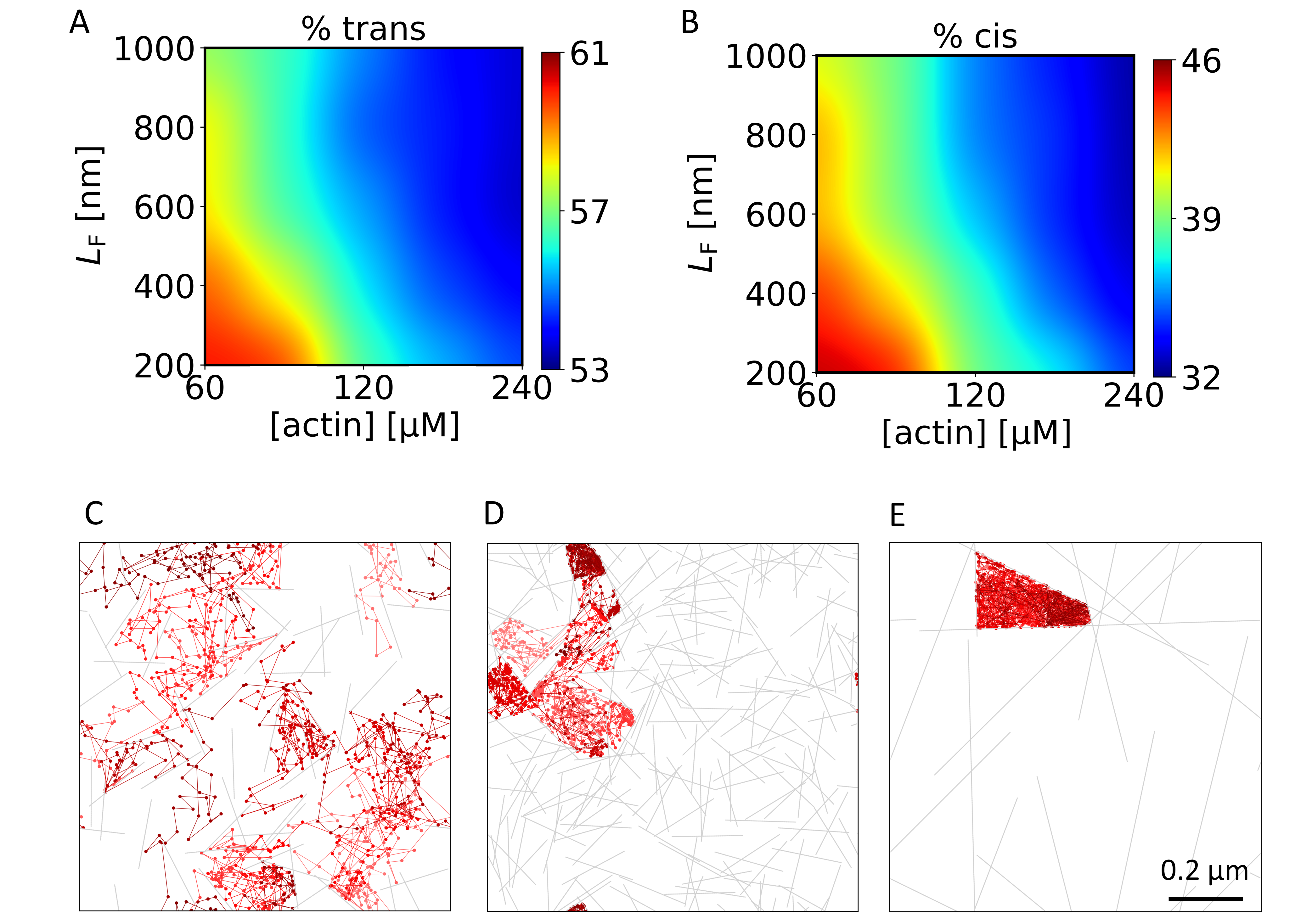

Supplement: S3 Fig — (A, B) trans and cis ratio for simulations in Fig 3. Each data point in heat maps is an average over 5 simulations. (C-E) Red dots and lines show the examples of cadherin trajectories under three conditions. Time point of the trajectory is indicated by the color scaling. Light red dots indicate early in the time point. Dark red dots indicate latter in the time point. Light gray lines represent actin filaments. (C) LF = 200 nm, [actin] = 60 μM, (D) LF = 200 nm, [actin] = 240 μM, and (E) LF = 1 μm, [actin] = 60 μM. Each data point in (A-B) represents the average of data obtained in 5 simulations performed under the same condition. (TIF) [file pcbi.1010257.s003.TIF]

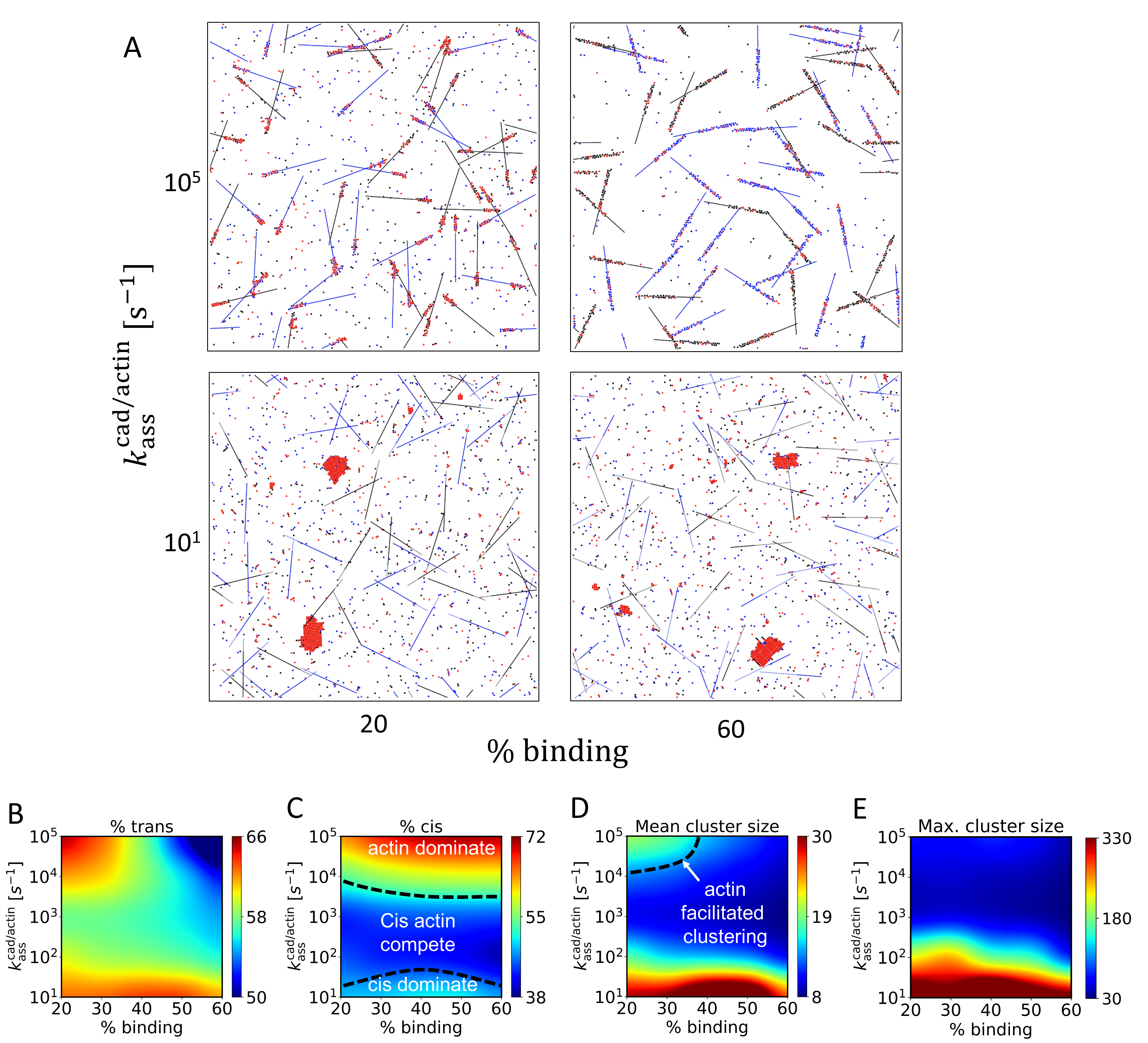

Supplement: S4 Fig — (A) Snapshots of simulations at 100 s with different F-actin concentration and filament length, kasscis=105s−1,kdiscis=103s−1, [cad]=1200μm−2,kdiscad/actin=10s−1. (B) The fraction of cadherin molecules existing as trans dimer. (C) The fraction of cadherin molecules involved in cis interactions. (D) Mean cadherin cluster size for clusters with more than 4 monomers. (E) Maximum cluster size. (TIF) [file pcbi.1010257.s004.TIF]

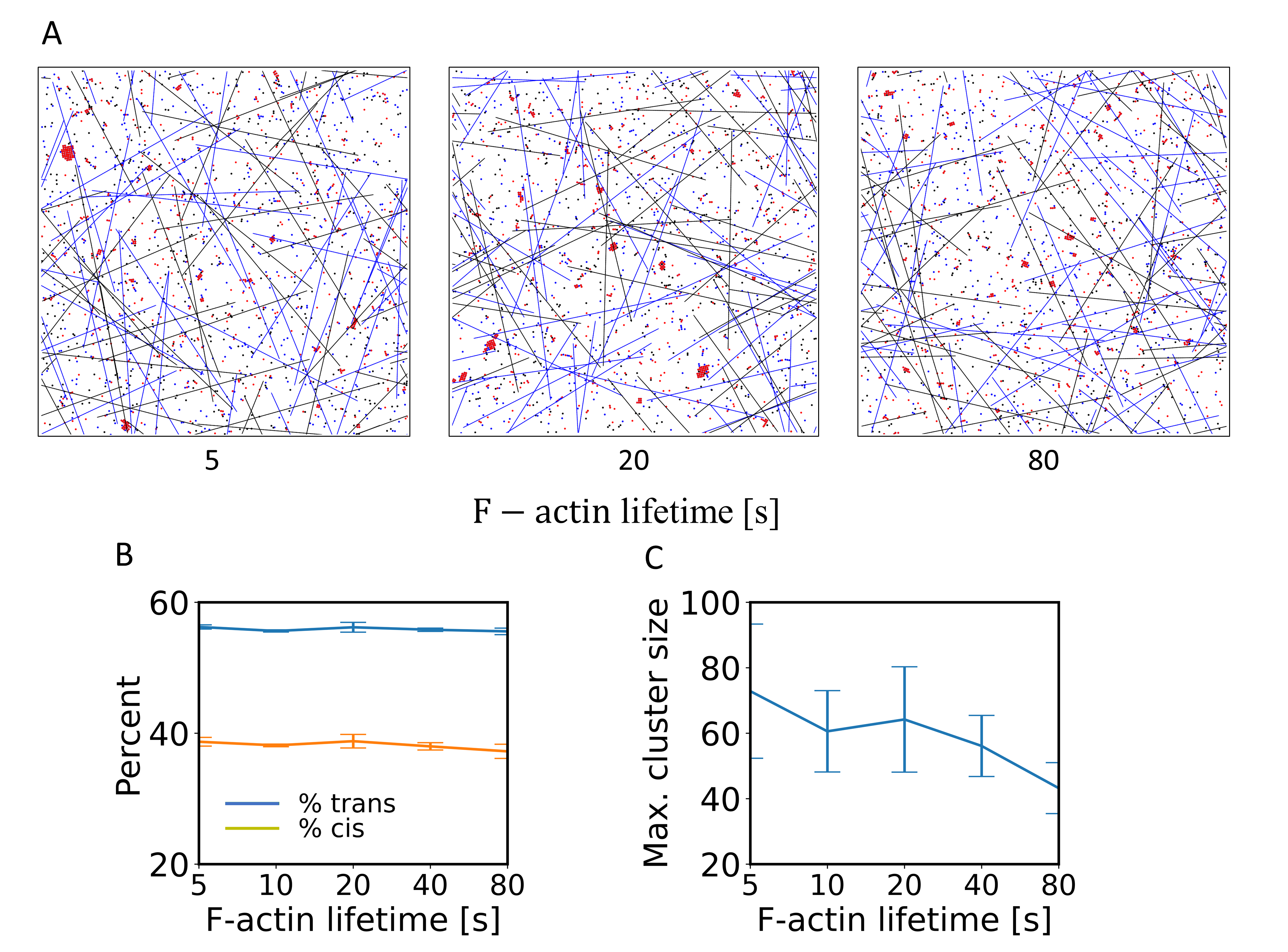

Supplement: S5 Fig — (A) Snapshots of simulations at 100 s, kasscis=105s−1,kdiscis=103s−1,[cad]=1200μm−2, Lf = 600 μm, [actin] = 120 μM. Binding between cadherin and F-actin is disabled. (B) The fraction of cadherin molecules existing as trans dimer (green curve) and the fraction of cadherin molecules involved in cis interactions (red curve). (C) Maximum cluster size at 100s. Each curve in (B-C) represents the average of data obtained in 5 simulations performed under the same condition. (TIF) [file pcbi.1010257.s005.TIF]
