# Supplementary material for: Role of actin filaments and cis binding in cadherin clustering and patterning
Source: PLoS Comput Biol. 2022 Jul 8;18(7):e1010257. doi: 10.1371/journal.pcbi.1010257 (PMC9299298; doi:10.1371/journal.pcbi.1010257)
Supplement: S1 Table — The value of dccad/actin equals half of the diameter of actin filaments [41]. The values of ϕF−actin/memb were selected to cover a wide range of actin orientations. The range of kasscad/actin is determined arbitrarily due to lack of references. (DOCX) [file pcbi.1010257.s006.docx]

**S1 Table. Simulations Parameters**

| Parameter | Description | Values | Reference |
| --- | --- | --- | --- |
| $\boldsymbol{d}_{\mathbf{c}}^{\mathbf{trans}}$ | Cutoff distance for trans dimerization | $1.5 nm$ | [1] |
| $\boldsymbol{d}_{\mathbf{c}}^{\mathbf{cis}}$ | Cutoff distance for cis interaction | $3 nm$ | [1] |
| $\boldsymbol{\theta}^{\mathbf{trans}}$ | Packing angle between monomers in trans dimer | $90 ֯$ | [1] |
| $\boldsymbol{\theta}^{\mathbf{cis}}$ | Packing angle between monomers in cis dimer | $180 ֯$ | [1] |
| $\boldsymbol{D}^{\mathbf{cadherin}}$ | 2D diffusion coefficient of a cadherin monomer | $28*{10}^{-3} \mu m^{2}s^{-1}$ | [2] |
| $\boldsymbol{r}^{\mathbf{cadherin}}$ | Radius of a cadherin rigid body | $2.5 nm$ | [1] |
| $\boldsymbol{d}_{\mathbf{c}}^{\mathbf{cad/actin}}$ | Cutoff distance for cadherin F-actin binding | $3.5 nm$ | Estimation |
| $\boldsymbol{L}_{\mathbf{F}}$ | Length of an actin filament | $200-1000 nm$ | [3, 4] |
| $\boldsymbol{T}_{\boldsymbol{actin}}$ | Thickness of cadherin effective region | $50-100 nm$ | [5] |
| $\boldsymbol{\phi}^{\mathbf{F-actin/memb}}$ | Angle between actin filament and the membrane | $15 ֯-75 ֯$ |  |
| $\boldsymbol{k}^{\mathbf{M-X}}$ | Forward rate constant of binding from M-M to X | $3.8*{10}^{4} s^{-1}$ | [6] |
| $\boldsymbol{k}^{\mathbf{X-M}}$ | Backward rate constant of binding from X to M-M | $1.84*{10}^{3} s^{-1}$ | [6] |
| $\boldsymbol{k}^{\mathbf{M-S}}$ | Forward rate constant of binding from M-M to S | $3.1*{10}^{-1} s^{-1}$ | [6] |
| $\boldsymbol{k}^{\mathbf{S-M}}$ | Backward rate constant of binding from S to M-M | $1.27*{10}^{-4} s^{-1}$ | [6] |
| $\boldsymbol{k}^{\mathbf{X-S}}$ | Forward rate constant of binding from X to S | $86 s^{-1}$ | [6] |
| $\boldsymbol{k}^{\mathbf{S-X}}$ | Backward rate constant of binding from S to X | $0.8 s^{-1}$ | [6] |
| $\boldsymbol{k}_{\mathbf{ass}}^{\mathbf{cis}}$ | Intrinsic cis association rate | ${10}^{1}-{10}^{5} s^{-1}$ | [1] |
| $\boldsymbol{k}_{\mathbf{dis}}^{\mathbf{cis}}$ | Intrinsic cis dissociation rate | ${10}^{-2}-{10}^{3} s^{-1}$ | [1] |
| $\boldsymbol{k}_{\mathbf{ass}}^{\mathbf{cad/actin}}$ | Cadherin F-actin association rate | ${10}^{1}-{10}^{5} s^{-1}$ | Estimation |
| $\boldsymbol{k}_{\mathbf{dis}}^{\mathbf{cad/actin}}$ | Cadherin F-actin dissociation rate | $0.1 \& 10 s^{-1}$ | [7, 8] |

**S1 Table. Outlines all the parameters and associated references used in the computational model.** The value of $\boldsymbol{d}_{\mathbf{c}}^{\mathbf{cad/actin}}$ equals half of the diameter of actin filaments [9]. The values of $\boldsymbol{\phi}^{\mathbf{F-actin/memb}}$ were selected to cover a wide range of actin orientations. The range of $\boldsymbol{k}_{\mathbf{ass}}^{\mathbf{cad/actin}}$ is determined arbitrarily due to lack of references.

**REFERENCES**

1. Chen J, Newhall J, Xie ZR, Leckband D, Wu Y. A Computational Model for Kinetic Studies of Cadherin Binding and Clustering. Biophys J. 2016;111(7):1507-18. doi: 10.1016/j.bpj.2016.08.038. PubMed PMID: 27705773; PubMed Central PMCID: PMCPMC5052510.

2. Iino R, Koyama I, Kusumi A. Single molecule imaging of green fluorescent proteins in living cells: E-cadherin forms oligomers on the free cell surface. Biophys J. 2001;80(6):2667-77. doi: 10.1016/S0006-3495(01)76236-4. PubMed PMID: 11371443; PubMed Central PMCID: PMCPMC1301454.

3. Vinzenz M, Nemethova M, Schur F, Mueller J, Narita A, Urban E, et al. Actin branching in the initiation and maintenance of lamellipodia. J Cell Sci. 2012;125(Pt 11):2775-85. Epub 2012/03/21. doi: 10.1242/jcs.107623. PubMed PMID: 22431015.

4. Kasza KE, Broedersz CP, Koenderink GH, Lin YC, Messner W, Millman EA, et al. Actin filament length tunes elasticity of flexibly cross-linked actin networks. Biophys J. 2010;99(4):1091-100. Epub 2010/08/18. doi: 10.1016/j.bpj.2010.06.025. PubMed PMID: 20712992; PubMed Central PMCID: PMCPMC2920742.

5. Bertocchi C, Wang Y, Ravasio A, Hara Y, Wu Y, Sailov T, et al. Nanoscale architecture of cadherin-based cell adhesions. Nat Cell Biol. 2017;19(1):28-37. doi: 10.1038/ncb3456. PubMed PMID: 27992406; PubMed Central PMCID: PMCPMC5421576.

6. Li Y, Altorelli NL, Bahna F, Honig B, Shapiro L, Palmer AG, 3rd. Mechanism of E-cadherin dimerization probed by NMR relaxation dispersion. Proc Natl Acad Sci U S A. 2013;110(41):16462-7. doi: 10.1073/pnas.1314303110. PubMed PMID: 24067646; PubMed Central PMCID: PMCPMC3799306.

7. Huang DL, Bax NA, Buckley CD, Weis WI, Dunn AR. Vinculin forms a directionally asymmetric catch bond with F-actin. Science. 2017;357(6352):703-6. doi: 10.1126/science.aan2556. PubMed PMID: 28818948; PubMed Central PMCID: PMCPMC5821505.

8. Buckley CD, Tan J, Anderson KL, Hanein D, Volkmann N, Weis WI, et al. Cell adhesion. The minimal cadherin-catenin complex binds to actin filaments under force. Science. 2014;346(6209):1254211. doi: 10.1126/science.1254211. PubMed PMID: 25359979; PubMed Central PMCID: PMCPMC4364042.

9. Cooper. GM. The Cell: A Molecular Approach. 2nd edition. Sunderland (MA): Sinauer Associates; 2000. Structure and Organization of Actin Filaments. Available from: <https://www.ncbi.nlm.nih.gov/books/NBK9908/2000>.
